# Non-necrotizing and necrotizing soft tissue infections in South America: A retrospective cohort study

**DOI:** 10.1016/j.amsu.2020.09.013

**Published:** 2020-09-11

**Authors:** Gustavo Lopes Gomes Siqueira, Ricardo Alves de Olinda, Camila Meira Barbosa de Siqueira, Analice Barros de Vasconcelos Sá Torres, Luana de Carvalho Viana Corrêa, Francisco de Assis Silva Lacerda, Pablo Luiz Fernandes Guimarães

**Affiliations:** a Clinical Surgery Department, Faculty of Medical Sciences, UNIFACISA, Campina Grande, Paraiba, Brazil; b Department of Medical Statistics, State University of Paraiba, UEPB, Paraiba, Brazil; c Nurse Department, Federal University of Campina Grande, UFCG, Paraiba, Brazil; d UNIFACISA, Campina Grande, Paraiba, Brazil

**Keywords:** Soft tissue infection, Necrotizing fasciitis, Lymphangitis, Erysipelas

## Abstract

**Background:**

This study analyzed and described factors related to necrotizing or non-necrotizing soft tissue infections (SSTIs) in a hospitalized patient population in Northeastern South America.

**Materials and methods:**

This retrospective study included patients hospitalized with SSTIs between January 2011 and December 2016. The main factors related to necrotizing SSTIs (NSTIs) or non-necrotizing SSTIs were analyzed together or separately.

**Results:**

Of 344 SSTI patients (161 [46.8%] non-necrotizing, 183 [53.2%] necrotizing), NSTI patients had a higher incidence of heart disease (*P* = 0.0081) and peripheral arterial disease (PAD; *p* < 0.001), more antibiotic use, and longer hospital stay (*P* < 0.001). NSTI was associated with a 9.58, 33.28, 2.34, and 2.27 times higher risk of PAD (confidence interval [CI] 3.69–24.87), amputation (7.97–139), complications (1.45–3.79), and death (1.2–4.26), respectively, than non-necrotizing SSTI. The risk factors associated with amputation were PAD (*P* < 0.001) and poor glycemic control during hospitalization (*P* = 0.0011). Factors associated with higher mortality were heart disease (*P* < 0.001), smoking (*P* = 0.0135), PAD (*P* = 0.001), chronic renal failure (*P* = 0.0039), poor glycemic control (*P* = 0.0005), and evolution to limb irreversibility (*P* < 0.001).

**Conclusion:**

Patients with NSTI have greater illness severity, with a greater association with PAD and amputation. Patients with poor glycemic control more frequently underwent amputation and died.

## Highlights

• Patients with necrotizing infections and poor glycemic control have higher risks for amputation.
• Diabetes as a risk factor most associated with soft tissue infections.
• Peripheral arterial disease is the main risk factor for amputation and death.
• Soft tissue infections with necrosis evolve with more complications and risk of amputation and death.

## 1 Introduction

Skin and soft tissue infections (SSTIs) constitute an inflammatory/infectious process that affects the skin and the underlying subcutaneous tissues and can extend to deeper tissues with variable severity. A recent meta-analysis on SSTIs did not include any articles from South America, although it included articles from all of the other continents [1]. The scientific interest in SSTIs has been neglected in comparison to that of other types of infections, although an important increasing incidence has been reported in the last decade, which is mainly due to resistant strains (methicillin-resistant *Staphylococcus Aureus* [MRSA]). Miller et al. reported an annual incidence of 48.5 cases per 1000 persons per year of SSTIs in the United States of, which is twice as high as the incidence of urinary tract infections and ten times higher than the incidence of pneumonia [2].

The main risk factors for SSTI are diabetes, hepatopathy, peripheral arterial disease (PAD), neutropenia, previous lower limb surgery (notably, with a saphenectomy strip), obesity, use of intravenous drugs, and chronic renal failure (CRF), among others [3,4] Several classifications have been published in the past decade, and were based principally on three elements: anatomical (infectious localization), depth, and etiological (pathogenic characteristics) features. The most important features of SSTIs are the depth and presence of necrosis, based on which they are classified as non-necrotizing SSTIs and necrotizing SSTIs (NSTIs) [[5], [6], [7]]. The most common examples of non-necrotizing infections are cellulitis and bullosa cellulitis. The signs of NSTI include erythematous swelling, uncomplicated demarcation of the wound edges, bullae (bullous cellulitis), and localized pain.

The presence of necrosis as a distinguishing feature of NSTI is an aspect that can be relatively easily identified, and the main patient characteristics are skin discoloration, regional numbness, pain disproportionate to the findings on physical examination, and indeterminable boundaries of the infection and tissue swelling [5]. NSTI can be classified by depth as: necrotizing cellulitis (affecting the dermis and subcutaneous tissues), and necrotizing fasciitis [7]. However, distinguishing between these infections can be very difficult; the gold standard in correctly diagnosing the infection is by gauging the extent of infection to the fascia on surgery [8]. Surgery remains the best option to diagnose NSTI and has the advantage that it is the specific treatment modality for NSTI. The importance of defining the type of NSTI emerges from the fact that the morbidity and mortality are higher when the fascia is afflicted, with more rapid disease progression, although the principles of treatment remain nearly the same: antimicrobial therapy and early surgery with resection of the necrotic and infected tissues [1,2,4]. The mortality rate of NSTI varies between 6% and 33%, and the amputation rate ranges from 12% to 20% [9]. Therefore, this study aimed to identify and describe the features of SSTIs in the Northeastern South American region, where despite the high morbidity and mortality associated with these infections, research on the subject is scarce.

## 2 Methods

This research is in line with the STROCSS criteria [10].

### 2.1 Study center

Our institution is an academic, 292-bed multispecialty medical emergency and trauma care referral center that provides healthcare services to a population of approximately 1.5 million people. It also includes a burn center. Approximately 8500 hospitalizations and nearly 800 surgeries are undertaken every month in this institution.

### 2.2 Study design

This study retrospectively reviewed the data of in-patients with SSTIs who were identified based on their electronic health records and who were treated from January 01, 2011 to December 31, 2016. Individuals with only lower limb SSTIs were eligible for inclusion in the study; we excluded patients with infections following critical limb ischemia (CLI), such as toe necrosis or any other necrosis of the feet. Moreover, we excluded patients with Fournier gangrene, pressure ulcers, surgical site infections, diabetic foot ulcers, and unknown diagnoses. This study was approved by the ethics committee of the institution.

Based on the options marked in the medical records following daily evaluations, the diagnosis was subdivided in four categories: (1) cellulitis (simple); (2) bullous cellulitis; (3) necrotizing cellulitis; and (4) necrotizing fasciitis. Categories 1 and 2 were grouped as non-necrotizing SSTI and 3 and 4 as NSTI. The risk factors analyzed included: hypertension, diabetes, obesity (body mass index >30), heart disease, cirrhosis/alcoholism (history of more than 14 units of 12 g for men and 7 for women), smoking, peripheral arterial disease (PAD; absence of peripheral pulse or ankle–brachial index <0.9), and chronic renal failure (CRF). The laboratory findings at the time of admission, which included leukocytosis and white blood cell count (WBC), glycemic values, and glycemic control (uncontrolled if mean average was >250 mg/dL during hospitalization). Regarding treatments and outcomes, we evaluated the length of hospital stay (days), period of the entrance (regarding weather stations), number of antimicrobial agents and duration of therapy, number of debridement procedures, major amputation due to infection, complications, and mortality. We also analyzed the specific weather for the time of year that the patient was admitted.

### 2.3 Data analysis

The differences between the two groups with respect to diagnostic categories and the demographic risk factors, laboratory findings, surgical interventions, and outcome parameters were assessed using the chi-square adherence test, Fisher exact test, and Kruskal–Wallis test, as appropriate. After aggregating the diagnoses of NSTI and non-necrotizing SSTI, we used multiple regression analysis to obtain the risk ratio (RR) between independent factors. Differences with *P* < 0.05 were considered significant.

## 3 Results

A total of 344 patients with SSTIs were included in the study: 11 (3.2%) cellulitis patients; 150 (43.6%) bullous cellulitis; 169 (49.1%) necrotizing cellulitis; and 14 (4.1%) necrotizing fasciitis patients. Demographic characteristics and risk factors are shown in Table 1, and hypertension (64.4%) and diabetes (70.1%) were the commonest comorbidities. Among the other risk factors evaluated, obesity (12.2%), heart disease (17.4%), hepatopathy (6.1%), smoking (8.7%), PAD (14%), and CRF (7.6%) were not detected in the majority of the patients. An analysis of the risk factors by the diagnoses showed that heart disease (*P* = 0.0081) and PAD (*P* < 0.001) were most commonly associated with NSTIs in the study population (Table 2).

**Table 1:** Demographic characteristics and severity of illness in the study population.

	SSTI		Complications		Death	
	N (%)	X^2^a^ *	N (%)	*P*-value^b^	N (%)	*P*-value^b^
Hypertension	232 (67.4)	41.86	80 (74.8)	0.0682	41 (77.4)	0.0939
Diabetes	241 (70.1)	55.36	76 (71.0)	0.7919	41 (77.4)	0.2071
Obesity	42 (12.2)	46.72	11 (10.3)	0.5938	11 (7.5)	0.3617
Heart Disease	60 (17.4)	145.86	43 (40.2)	<0.001	26 (49.1)	**<0.001**
Hepatopathy	21 (6.1)	265.13	14 (13.1)	0.0008	6 (11.3)	0.1117
Smoking	30 (8.7)	234.47	18 (13.1)	0.0007	10 (18.9)	**0.0135**
PAD	48 (14.0)	234.47	23 (21.5)	0.0067	15 (28.3)	**<0.001**
CRF	26 (7.6)	246.88	12 (11.3)	0.1198	8 (15.4)	**0.0399**

PAD, peripheral arterial disease; CRF, chronic renal failure.

*P-value <0.001.

Bold values indicate significant differences.

a Chi-square adherence test.

b Fisher test.

**Table 2:** Clinical features of the patients in the two study groups with skin and soft tissue infection (SSTI).

		Non-necrotizing		Necrotizing		*P*-value
		Cellulitis (N = 11)	Bullous cellulitis (N = 150)	Cellulitis (N = 169)	Fasciitis (N = 14)	
Sex	Female (N = 204)	8 (72.7%)	90 (60%)	98 (58.3%)	8 (57.1%)	0.8572^a^
	Male (N = 139)	3 (27%)	60 (40%)	70 (41.7%)	6 (42.9%)	
Age, years		62^b^	66	70	75	0.1138^d^
Hypertension (N = 232)		7	93	122	10	0.2556^a^
Diabetes (N = 241)		7	98	127	9	0.2206^a^
Obesity		2	17	21	2	0.1824
Heart disease (N = 60)		0	19 (12.7%)	35 (20.7%)	6 (42.9%)	**0.0081**^a^
Hepatopathy (N = 21)		0	8 (5.3%)	11 (6.5%)	2 (14.3%)	0.4442^a^
Smoking (N = 30)		0	11 (7.3%)	19 (11.2%)	0	0.3877^a^
PAD (N = 48)		0	5 (3.3%)	38 (22.5%)	5 (35.7%)	**<0.001**^a^
CRF (N = 26)		1 (9.1%)	10 (6.7%)	13 (7.7%)	2 (14,3%)	0.5205^a^
Hospitalization duration, days		4^b^ (3–7.5)^c^	8 (4–11)	8 (5–14)	11.5 (11–31)	**0.007**^d^
Number of antimicrobials		1.0^b^ (1–2)^c^	2.0 (2–2)	2.0 (2–2)	3.0 (1–4)	0.0108^d^
Duration of antimicrobial therapy		4^b^ (3–8)^c^	7.5 (4–11)	8 (5–14)	11.5 (10.2–20.25)	**<0.001**^d^
White blood cell count at admission, per mm³		11.5^b^ (9–12.7)^c^	139 (10.0–20.5)	14.9 (11.0–23.2)	16.7 (19.0–31.2)	**0.0022**^d^
Glucose at admission, mg/dl		125^b^ (115–162)^c^	142 (112–203)	153 (109–269)	215 (145–187)	**0.0326**^d^

PAD, peripheral arterial disease; CRF, chronic renal failure.

Data are presented as n (%).

a Fisher test.

b Median.

c Interquartile range.

d Kruskal–Wallis test.

**Fig. 1:**
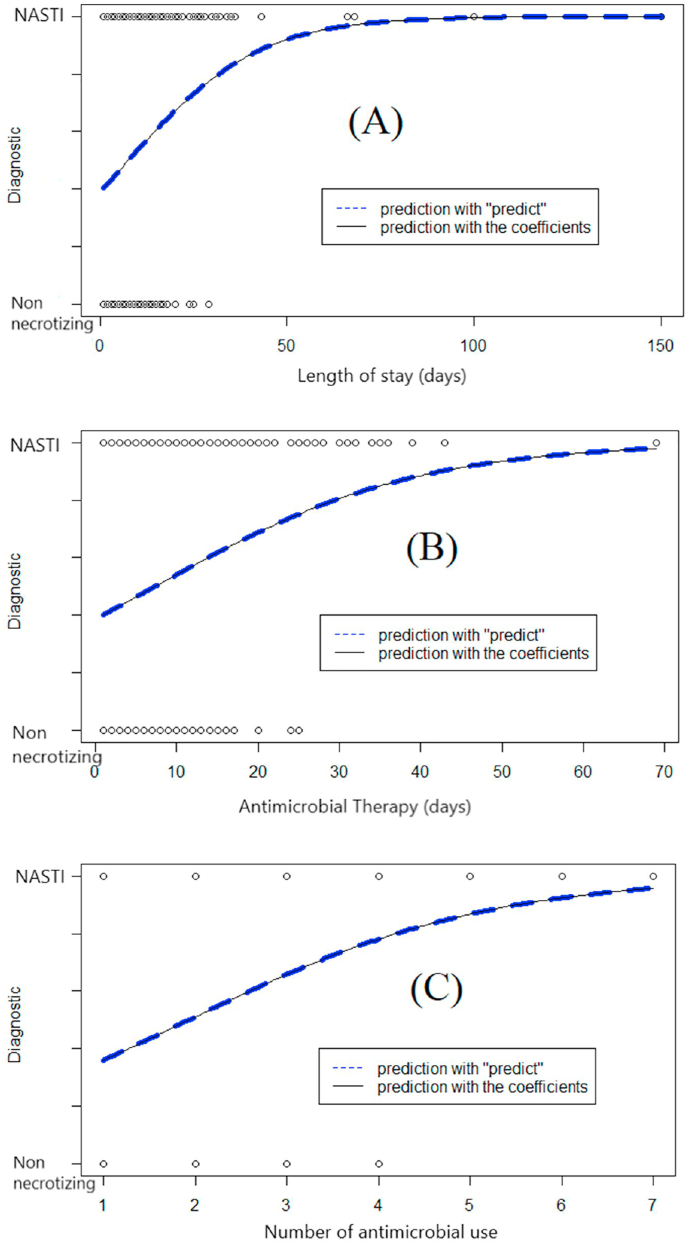
Tendency of continuous variables between the groups with non-necrotizing and necrotizing skin and soft tissue infection (SSTI). (A) Curve inclination for longer duration in necrotizing SSTI (NSTI); (B) inclination through longer duration of antimicrobial therapy in patients with NSTI; and (C) Tendency to use more antimicrobials in NSTI.

The mean ages of the patients were 59.6, 64.8, 68.9, and 67.1 years in the four different groups, respectively (Table 2). There were no significant differences among the diagnostic groups, but the NSTI group showed a tendency for age >60 years. An analysis of the relationship between the diagnosis and length of stay (days) showed mean durations of 5.82, 8.21, 10.66, and 18.00 days for cellulitis, bullous cellulitis, necrotizing cellulitis, and necrotizing fasciitis, respectively. Analysis of the median values (Table 2) showed an inclination (Fig. 1) for prolonged hospitalization among patients with NSTIs (*P* < 0.001).

The majority of the patients were female (204/344), although there were no statistically significant sex-based differences in the diagnoses. Moreover, there was no significant difference between the hospitalization duration and weather season variations (Table 2).

The evaluation of laboratory parameters showed that patients with NSTIs had higher (Table 2) median blood glucose levels at admission (*P* = 0.003). The WBC at admission showed a tendency to be higher in the NSTI group, with median values of 14.9 and 26.750 per mm^3^ in categories 3 and 4 of the diagnoses (*P* = 0.002). Poor glycemic control was associated with amputation (*P* = 0.001), a longer length of stay (*P* < 0.001), and was an important mortality risk factor (*P* = 0.0005); The institutional protocol precludes the use of imaging investigations, such as CT scanning or nuclear magnetic resonance, except in cases with diagnostic ambiguity. Therefore, there were no records of such imaging investigations in the medical records of the patients in this study.

The mean number of antimicrobial agents used was <2 for non-necrotizing SSTIs, which differed from 2.37 to 3.21 for categories 3 and 4 (NSTIs). The medians showed a tendency for a higher number of agents in NSTIs (Fig. 1; *P* = 0.0108). In 72.7% (8/11) of patients with simple cellulitis, crystalline penicillin was used as monotherapy. Patients in category 2 used ciprofloxacin (50.6% [76/150]) and clindamycin (62.6% [94/150]), with meropenem use in 13.6% (23/169) and vancomycin in 8.87% (15/169). In the necrotizing cellulitis group, 73.3% (129/169) and 64% (109/169) used clindamycin and ciprofloxacin, respectively. In the group with necrotizing fasciitis, meropenem and vancomycin was used in 92% (13/14) and 78% (11/14) of patients, respectively.

Surgical debridement was performed in 220 patients (Table 3) and indicated a higher tendency for necrotizing cellulitis (135/169 [79.9%]). The mean duration to submit the first surgery was 3.6, 2.3, and <1 day for groups 2, 3, and 4, respectively. We observed a higher trend for lower limb loss in NSTIs (*P* < 0.001; 2 patients (1.3%) with bullous cellulitis, 44 (26%) with necrotizing cellulitis, and 10 (71.4%) with necrotizing fasciitis). In category 3, 22 (13%) patients underwent a major amputation as first-line treatment, as soon as feasible and based on patient acceptance. Among the 14 patients with necrotizing fasciitis, 7 (50%) underwent broad debridement in the first 24 h (three of them worsened, with limb loss); 6 had unsalvageable limbs and underwent amputation as the first line of treatment.

**Table 3:** Surgical evaluation of the patients in the two study groups with skin and soft tissue infection (SSTI).

		Non-necrotizing SSTI		Necrotizing SSTI		*P*-value
		Cellulitis (N = 11)	Bullosa cellulitis (N = 150)	Necrotizing cellulitis (N = 169)	Necrotizing fasciitis (N = 14)	
Seasons	Spring (N = 94)	3 (27.3)	50 (33.3)	39 (23,1)	2 (14,3)	0,2864^a^
	Summer (N = 107)	4 (36.4)	47 (31.3)	54 (32.0)	2 (14.3)	
	Fall (N = 60)	1 (9.1)	22 (14.7)	33 (19.5)	4 (28.6)	
	Winter (N = 83)	3 (27.3)	31 (20.7)	43 (25.4)	6 (42.9)	
Debridement (N = 220)		1,0 (9.1)	77 (51.3)	135 (79.9)	7 (50.0)	**<0.001**^a^
Debridement (per patient)		0	1.0^b^ (0–1)^c^	1.0 (1–1)	0.5 (0–1)	**<0.001**^d^
Amputation (N = 56)		0	2 (1.3)	44 (26.0)	10 (71.4)	**<0.001**^a^
Complications (N = 107)		0	35 (23.3)	58 (34.3)	14 (100.0)	**<0.001**^a^
Death (N = 53)		0	16 (10.6)	30 (17.7)	7 (50.0)	**0.0014**^a^

Data are presented as n (%).

a Fisher test.

b Median.

c Interquartile range.

d Kruskal–Wallis test.

The analysis of the risk factors associated with amputation (Table 4) found one significantly associated factor: PAD (*P* < 0.001). An evaluation of outcome parameters showed that poor glycemic control is associated with higher risk of amputation (*P* = 0.0011). Neither diabetes nor obesity was significantly associated with this outcome. Analyzing patients with major amputations as the outcome showed a higher rate of complications and mortality (Table 6). The amputation rate was 29.5% (54/183) for NSTI patients versus 1.24% (2/161) in non-necrotizing SSTI patients.

**Table 4:** Factors associated with amputation.

		Amputation		
		Yes (N = 56)	No (288)	*P*-value^a^
Risk Factors	Hypertension (232)	40 (71.4)	192 (66.7)	0.4865
	Diabetes (242)	46 (82.1)	195 (67.7)	0.2206
	Obesity	11 (19,6)	49 (17)	0.3617
	Heart Disease (60)	18 (32.1)	42 (14.6)	0.7595
	Hepatopathy (21)	4 (7.1)	17 (5.9)	0.4442
	Smoking (30)	9 (16.1)	21 (7.3)	0.0649
	PAD (48)	34 (60,7)	14 (4.9)	**<0.001**
	CRF (26)	7 (12.7)	19 (6.6)	0.1582
Complications				
	Yes (107)	35 (62.5)	72 (25.0)	**<0.001**
	No (237)	21 (37.5)	216 (75.0)	
Death				
	Yes (53)	20 (35.7)	33 (11.5)	**<0.001**
	No (291)	36 (64.3)	255 (88.5)	

PAD, peripheral arterial disease; CRF, chronic renal failure.

Data are presented as n (%). Bold values indicate significant differences.

a Fisher test.

**Table 5:** Relationship of the clinical outcomes with the glycemic control.

		Glycemic status, controlled (N = 198)	Glycemic status, uncontrolled (N = 146)	
Diagnosis	Cellulitis, simple (N = 11)	9 (4.5)	2 (1.4)	**0.0254**^a^
	Bullosa cellulitis (N = 150)	93 (47.0)	57 (39.0)	
	Necrotizing cellulitis (N = 169)	92 (46.5)	77 (52.7)	
	Necrotizing fasciitis (N = 14)	4 (2.0)	10 (6.8)	
Complications	No (N = 237)	152 (76.8)	85 (58.2)	**0.0002**^a^
	Yes (N = 107)	46 (23.2)	61 (41.8)	
Amputation	No (N = 288)	177 (89.39)	111 (76.03)	**0.0011**^a^
	Yes (N = 56)	21 (10.60)	35 (23.97)	
Duration of hospitalization, days		7^b^ (4−11)^c^	9 (6–14)	**<0.001**^d^
Deaths	No (N = 291)	179 (90.4%)	112 (76.7%)	**0.0005**^a^
	Yes (N = 53)	19 (9.6%)	34 (23.3%)	

Data are presented as n (%). Bold values indicate significant differences.

a Fisher test.

b Median.

c Interquartile range.

d Kruskal–Wallis test.

**Table 6:** Risk ratio between necrotizing versus non-necrotizing skin and soft tissue infection.

Parameters		RR	95% CI	*P*-value
Laboratory	Leukocytosis	1.62	1.05–2.50	**0.0295**
	Glucose control: No vs Yes	1.55	1.00–2.39	**0.0418**
Risk Factors	Hypertension	1.58	1.00–2.49	**0.0480**
	Diabetes	1.54	0.97–2.45	**0.0670**
	Obesity	1,07	0,56–2,05	0,8280
	Heart disease	2.16	1.19–3.90	**0.0110**
	Hepatopathy	1.46	0.59–3.62	0.4120
	Smoking	1.58	0.73–3.43	0.2470
	PAD	**9.58**	3.69–24.87	**<0.001**
	CRF	1.22	0.55–2.75	0.6230
Evaluation	Duration of hospitalization, days	1.06	1.03–1.10	**<0.001**
	Debridement	**3.69**	2.31–5.87	**<0.001**
	Amputation	**33.28**	7.97–139.0	**<0.001**
	Complications	**2.34**	1.45–3.79	**0.0005**
	Death	**2.27**	1.2–4.26	**0.0097**

Bold values indicate significant differences. RR, risk ratio; CI, confidence interval; PAD, peripheral arterial disease; CRF, chronic renal failure.

Overall, 31.1% (107/344) of the patients evaluated with major complications and 15.4% (53/344) of the total study population died. We found that hepatopathy, heart disease, smoking, and PAD were associated with more complications, and heart disease, smoking, PAD, and CRF were associated with death (Table 1). In patients with NSTI, the complications rate was 39.3% (72/183), compared to 100% (14/14) in patients with necrotizing fasciitis. Complications in patients with non-necrotizing SSTI occurred at a rate of 21.3% and only in patients with bullous cellulitis. In total, we revealed a mortality rate of 15.4% (53/344); non-necrotizing SSTI was associated with death in 9.93% (16/161) of patients. Patients with NSTI had a mortality rate of 20.2% (37/183), which was 50% (7/14) in the necrotizing fasciitis group. Predictive factors of death (Table 1, Table 3, Table 4, Table 5) included heart disease (*P* < 0.001), smoking (*P* = 0.0135), PAD (*P* = 0.001), CRF (*P* = 0.0039), leukocytosis at admission (*P* = 0.0045), poor glycemic control (*P* = 0.0005), and indication for amputation (*P* < 0.001). Glycemic level at admission was not associated with complications or higher mortality.

The analysis indicated that there were 161 (46.8%) and 183 (53.2%) patients in the non-necrotizing SSTI and NSTI groups, respectively. A comparison of their risk factors (Table 6) showed a higher trend of diabetes, heart disease, and PAD in patients with NSTI, with mainly PAD showing an RR of 9.58 (confidence interval [CI] 3.69–24.87). Laboratory and outcome parameters in NSTI patients, for all parameters studied (Table 6), showed an RR of 33.28 (CI 7.97–139.0) of amputation (*p* < 0.001), 2.34 (CI 1.45–3.79) for complications (*P* = 0.0005), and 2.27 (CI 1.2–4.26) for mortality rate (*P* = 0.009). Regarding deaths, the mortality rates were 9.93% (16/161) in non-necrotizing SSTI and 20% (37/183) in NSTI.

## 4 Discussion

In the literature, SSTIs are commonly discussed, but NSTIs more rarely so. The proportion of NSTIs (53.2%) in our study population is higher than that reported in previous articles; Miller et al. reported a proportion of NSTIs of 23% among their SSTI patients [2]. The reason for the high proportion of NSTIs in this population could be that our institution is located in a region with a medium human development index (HDI), with an absolute value of 0.658 [11].

The reported global mean ages of >60 years and >65 years for NSTIs were higher than those reported previously, especially from developed countries [1,2,12] as Nawijn et al. reported in a meta-analysis (mean age of 54 years for NSTIs) [1]. Khamnuan et al. (2015) described a mean age of >60 years in a developing country (Thailand), generating a discussion on whether this factor influences NSTI prevalence [13].

In our study the most commonly associated risk factors were diabetes and heart disease, mainly for NSTIs, a finding which is in agreement with most of the literature [1,2,5,6,8]. Goh et al. identified diabetes as the overriding factor for NSTIs [14]. However, obesity was not an important risk factor in our study, which differs from the findings of other studies that considered it a major risk factor for the disease [2,3,6,12]. This difference is probably because the obesity index of the population we serve is 9% [15,16], which differs from the 40% and 29% reported for the US [17] and England [18], which are developed countries. In contrast, Tianyi et al. published a review of SSTIs in Africa and showed that the major risk factor for complicated SSTIs was obesity [19].

In the laboratory parameters, our institutional protocol includes only WBC, renal function, and glycemic control in hospitalized patients, reserving other exams on need-based requirement because of a limited budget. We agree with Jenkins et al. that a substantial amount of healthcare resources is expended on diagnostic tests, some of which may have been avoidable [20,21]. Several scores have already been studied, but there is a lack of consistent evidence in the diagnosis and prognosis of patients [22]. Leukocytosis does not generally have a major role in researching the evolution of the disease [22], although our study found cases of necrotizing infection had high tendencies for elevated WBC counts and high glucose levels at admission, including a median WBC count of 26750/mm^3^ (19225–31200) for necrotizing fasciitis. In addition, we found that poor glycemic control was an important risk factor for longer hospitalization and higher rates of complications and deaths. Most studies compare more reliable severity scores to predict outcomes [23], although strict glycemic control can be an important ally in combating complications from SSTIs. In general, diabetic patients with blood glucose levels <200 mg/dL are easier to treat [24]. Nonspecific studies for SSTI have described that uncontrolled blood glucose is a major risk factor for amputation [25].

The expected results were obtained with a tendency for longer hospitalizations and a greater number of antibiotics in NSTI patients. The institution has a protocol for admitting patients with necrotizing infections and using broad-spectrum antibiotics for polymicrobial infections. In non-necrotizing infections, we do not routinely use agents with efficacy against MRSA, in line with some studies that indicated that these do not improve results [26,27].

With regard to surgery, an average of 1.08 debridement procedures was done for patients diagnosed with necrotizing cellulitis and less than one with a diagnosis of necrotizing fasciitis, in contrast to that reported previously, with an average of three debridement procedures per patient [12,28]. This is probably because 13% of the patients with necrotizing cellulitis and 42.8% of those with necrotizing fasciitis underwent major amputation as the first line of treatment. Two main factors may be responsible for these numbers: *Doctor delay* and *System delay*. During the study period, the institution did not have an efficient system for regulating beds, being a referral center for cities within a radius of up to 350 km, which greatly delays the transfer and evaluation of the patient. Because the incidence is low, necrotizing infections can be confused for non-necrotizing infections, like simple cellulitis and erysipelas, in the crucial initial stages of treatment [2,28]. We believe that due to an inefficient regulatory system, many patients do not receive necessary care, causing their conditions to deteriorate; several high-severity patients with an initial indication for amputation were admitted during the study. The amputation rate was 29.5% for patients with NSTIs and 1.24% for non-necrotizing SSTIs, which is higher than the average reported by other studies (10–26%) [[29], [30], [31]].

In an analysis of risk factors, we found only PAD and glycemic control were major predictive factors for amputation in NSTIs. Most studies report the presence of heart disease, necrosis, serum creatinine >1.6 mg/dL, the presence of shock at admission, and anaerobic infections as main risk factors [13,32]. Although diabetes can be considered a risk factor for amputation [1,31], some studies have not found such an association [13,[33], [34], [35]].

A literature review demonstrated a complication rate of 55–59% in patients [1,2,12,13], with the same risk factors for mortality: advancing age, multiple comorbidities, sepsis on admission, and signs of circulatory shock [1,36,37]. The percentage of complications in our study was in agreement with that in the literature, as was our mortality rate of 21.3%. One recent meta-analysis [1] described a constant mortality rate in the last 10 years of approximately 20%, after a significant increase in the first few years of the 21st century, when it was identified that this increase was due to resistant microorganisms [2]. We studied the factors associated with mortality, and identified that leukocytosis on entry, uncontrolled blood glucose levels during hospitalization, and amputation were associated with higher mortality. The factors that seem to significantly improve the mortality in patients with NSTI include broad-spectrum antibiotic therapy on admission and ample debridement surgery in the first 12 h, although surgery in the first 24 h has not shown an improvement in amputation rates [1].

A challenge in evaluating the literature arises because there is no universally accepted standard of classification for SSTIs. Variations in the presence or absence of purulent secretion, necrosis or systemic complications have been described [38]. We believe that the presence or absence of necrosis facilitates the classification, and the use of different terms may delay treatment. Raya-Cruz et al. (2014) reported a study on SSTIs in 2014 with 66.7% of cases hospitalized with cellulitis/erysipelas, with the commonest risk factors being diabetes (33%) and heart disease (17.7%) [39]. Our study found an incidence of 46.8% for cellulitis/erysipelas, with diabetes in 65.2% (105/161) of those patients, which can be explained by the socioeconomic factors of our region, which contribute to less preventive glycemic control.

We compared NSTI with non-necrotizing SSTI, and a higher proportion of patients with diabetes, heart disease, and PAD had necrosis (PAD, RR 9.98, CI 3.69–24.87). The presence of necrosis proved to be an important illness-severity factor, with higher RR for leukocytosis, uncontrolled blood glucose, number of agents and duration of antibiotic therapy, and longer hospital stay, despite the latter having only a small increase. Patients with necrosis were three times more likely (RR 3.69, CI 2.31–5.87) to undergo debridement surgery and also demonstrated an important difference in the chance of being subjected to greater amputation (RR 33.28, CI 7.97–139.0). With these data, special attention needs to be paid to the prevention of necrotizing infections, especially in patients with PAD, as it has a greater than twofold RR for evolving into complications (RR 2.34, CI 1.45–3.79) and death (RR 2.27, CI 1.20–4.26).

The main limitation of this study lies in the fact that it is retrospective. We faced difficulties in comparing our data with published data from the same region due to the scarcity of studies on the subject in our continent. There is a need for prospective research in this geographic region and continent, due to socioeconomic and genetic differences that are evident in studies reported from other regions.

## 5 Conclusions

In this study region, patients with SSTIs had a higher mean age and lower proportion of obesity, and an equivalent presence of factors such as diabetes, heart disease, and PAD in comparison to the incidence reported in international literature. There was a higher overall amputation rate, despite a similar mortality rate, in the study population. PAD and glycemic control were most frequently associated with amputation. Comparing the presence or absence of necrosis showed a trend for greater severity in necrotizing infections, with an important increase in the risk of amputation, complications, and mortality.

## Provenance and peer review

Not commissioned, externally peer reviewed.

## Ethical approval

The study was approval by the ethical committee of the Emergency and Urgency Hospital Dom Luis Gonzaga, signed by the technical director Dr. Gilney Silva Porto number 305.794–1.

## Consent

We didn't use any issue that showed identifying of the patients, as is retrospective.

## Registration of research studies

1. Name of the registry: Comparison between non-necrotizing and necrotizing skin and soft tissue infections in northeastern South America: a retrospective comparative analysis.

2. Unique Identifying number or registration ID: researchregistry5796.

3. Hyperlink to your specific registration (must be publicly accessible and will be checked): https://www.researchregistry.com/browse-theregistry#home/registrationdetails/5f088eb265238a00155b4d4b/

## Declaration of competing interest

None.

## Study design

We made a cohort comparative study between non-necrotizing and necrotizing soft-tissue infections of lower extremities.
